# Molecular and neural bases underlying roles of BDNF in the control of body weight

**DOI:** 10.3389/fnins.2013.00037

**Published:** 2013-03-21

**Authors:** Filip Vanevski, Baoji Xu

**Affiliations:** Department of Pharmacology and Physiology, Georgetown University Medical CenterWashington, DC, USA

**Keywords:** brain-derived neurotrophic factor, TrkB, obesity, energy expenditure, ventromedial hypothalamus, paraventricular hypothalamus, dorsal vagal complex

## Abstract

Brain-derived neurotrophic factor (BDNF) is a potent regulator of neuronal development and synaptic plasticity that is fundamental to neural circuit formation and cognition. It is also involved in the control of appetite and body weight, with mutations in the genes for BDNF and its receptor, TrkB, resulting in remarkable hyperphagia and severe obesity in humans and mice. Recent studies have made significant progress in elucidating the source, action sites, and regulatory pathways of BDNF with regard to its role in the control of energy homeostasis, and have shed light on the relationships between BDNF and other molecules involved in the control of body weight. Here we provide a comprehensive review of evidence from pharmacological, genetic, and mechanistic studies, linking BDNF to the control of body weight. This review also aims to organize the main findings on this subject into a more refined framework and to discuss the future research directions necessary to advance the field.

## Introduction

Brain-derived neurotrophic factor (BDNF) is a member of the neurotrophin family of secreted signaling molecules that includes nerve growth factor (NGF), neurotrophin-3 (NT3), and neurotrophin-4/5 (NT4/5). Signal transduction can occur through the binding of two distinct classes of receptor proteins: the tropomyosin receptor kinase (Trk) family of receptor tyrosine kinases, which includes TrkA, TrkB, and TrkC, or the p75 neurotrophin receptor (p75^NTR^). The Trk receptors have different neurotrophins as their preferred ligands, with NGF binding to TrkA, BDNF and NT4/5 binding to TrkB and NT3 binding to TrkC, while all of the neurotrophins can bind to p75^NTR^ (Barbacid, 1994; Bibel and Barde, 2000; Reichardt, 2006). Neurotrophins are expressed both during development and throughout adulthood (Hofer et al., 1990; Yan et al., 1997b). They play crucial roles both in the proper wiring of neural circuits and in the modulation of connections in the mature nervous system by influencing neuronal survival and differentiation as well as synapse formation and plasticity (Chao, 2003; Waterhouse and Xu, 2009).

Perturbation of normal BDNF levels has been associated with many disease states, including Alzheimer's disease, Parkinson's disease, Huntington's disease, schizophrenia, and depression (Teixeira et al., 2010; Diniz and Teixeira, 2011; Satomura et al., 2011). Recent genetic data has also linked deficiencies in TrkB signaling to obesity (Rios et al., 2001; Xu et al., 2003; Yeo et al., 2004).

A key molecule involved in the control of body weight is leptin, which is the protein product of the *ob* gene and is produced and secreted from adipose tissue, and serves as an indicator of fat mass (Zhang et al., 1994; Elmquist et al., 1998). Its expression and secretion was found to be increased after either food intake or insulin injection, indicating that in addition to serving as a measure of adiposity, leptin levels can be modulated by nutritional status, probably through changes in insulin release regulated by blood glucose levels (Saladin et al., 1995; Barr et al., 1997; Boden et al., 1997). Treatment with leptin can lead to reduced fat storage through reduction in food intake as well as by increasing energy expenditure (Hamann and Matthaei, 1996; Stehling et al., 1996). In addition to leptin, many other factors have been shown to regulate energy balance, including insulin produced in the pancreas (Woods et al., 1979), glucagon-like-polypeptide-1 produced in the ileum (Tang-Christensen et al., 1996; Turton et al., 1996), peptide tyrosine tyrosine produced in the terminal ileum and colon (Batterham et al., 2002), cholecystokinin (CCK) produced in the duodenum (Dockray, 2012), ghrelin produced in the stomach (Kojima et al., 1999; Nakazato et al., 2001), and various metabolites such as glucose, fatty acids, and amino acids (Loftus et al., 2000; Cota et al., 2006; He et al., 2006; Pocai et al., 2006).

These peripheral factors reach the brain via the circulatory system and are integrated in several brain regions, including the arcuate nucleus (ARC), dorsomedial hypothalamus (DMH), ventromedial hypothalamus (VMH), paraventricular hypothalamus (PVH), and brainstem (Flier, 2004; Morton et al., 2006). The ARC is a hypothalamic nucleus with a well-demonstrated role in responding to peripheral signals of nutritional status, such as leptin, to regulate energy balance (Coppari et al., 2005). There are two distinct populations of neurons in the ARC involved in mediating this response: one that co-expresses proopiomelanocortin (POMC) and cocaine- and amphetamine-regulated transcript (CART), while the other co-expresses agouti-related protein (AgRP) and neuropeptide Y (NPY) (Parker and Bloom, 2012). One metabolite of POMC, alpha melanocyte-stimulating hormone (α-MSH), is an agonist of the melanocortin 4 receptor (MC4R). Conversely, AgRP is an inverse agonist of the MC4R (Ollmann et al., 1997). Furthermore, NPY can activate its receptors on POMC neurons in the ARC to induce hyperpolarization and decrease excitability (Cowley et al., 2001). Both populations express the leptin receptor, with signaling in POMC/CART neurons leading to increased secretion of α-MSH and activation of anorexigenic pathways, while signaling in AgRP/NPY neurons results in decreased secretion of AgRP and NPY and relief of inhibition of these pathways (Ellacott and Cone, 2006; Williams et al., 2011). POMC neurons in the ARC receive strong excitatory input from the medial VMH, which is decreased during fasting. NPY neurons in the ARC only receive weak inhibitory input from within the ARC itself (Sternson et al., 2005).

## The *Bdnf* gene and its expression

The mouse and rat *Bdnf* genes have nine exons, the first eight of which contain 5′ untranslated regions (5′ UTRs), with exon IX containing the entire coding sequence as well as the 3′ untranslated region (3′ UTR) (Timmusk et al., 1993; Aid et al., 2007). Transcription can be initiated at any of the eight untranslated exons, which are spliced to the common coding exon IX, generating mRNA species with different 5′ UTRs that code for the same protein. The number of potential transcripts is further increased by polyadenylation at either of two sites, one 350 bases downstream of the stop codon and the other at 2.85 kb downstream. Although the various transcripts encode the same protein, they are expressed differentially throughout development, have distinct spatial expression patterns, and respond uniquely to stimulation. Synthesis of some of these transcripts is regulated by the activity of the cyclic AMP responsive element binding protein (CREB) and methyl CpG-binding protein 2 (Tao et al., 1998; Chen et al., 2003). Recent studies investigating the relative importance of *Bdnf* mRNA sequences in directing mRNA trafficking have led to conflicting reports of which regions are involved, with work in our laboratory demonstrating a critical role for the long *Bdnf* 3′ UTR in directing dendritic trafficking (An et al., 2008), while another lab has shown the importance of coding and 5′ UTR sequences (Chiaruttini et al., 2009; Baj et al., 2011). The various temporal, spatial and activity-dependent patterns of transcription for the different *Bdnf* mRNA species allows for highly specific and fine-tuned regulation of BDNF synthesis, which can be further refined by post-transcriptional regulatory mechanisms acting on RNA transcript stability and translational activation.

*Bdnf* mRNA has been detected in many brain regions, including the cortex, hippocampus, amygdala, DMH, PVH, VMH, lateral hypothalamus (LH), ventral tegmental area (VTA), substantia nigra and nucleus of the solitary tract (NTS) (Conner et al., 1997; Xu et al., 2003). BDNF immunoreactive fibers, indicating possible sites of BDNF/TrkB signaling, were found in the cortex, hippocampus, nucleus accumbens (NAc), amygdala, ARC, DMH, LH, PVH, VMH, VTA, substantia nigra, dorsal raphe nucleus, and NTS (Conner et al., 1997; Katoh-Semba et al., 1997; Yan et al., 1997b). TrkB in the adult rat brain has been localized to the olfactory system, cortex, hippocampus, most nuclei of the hypothalamus, striatum, amygdala, septal nuclei, substantia nigra, cerebellar Purkinje cells, brainstem and spinal motor neurons, and brainstem sensory nuclei (Masana et al., 1993; Yan et al., 1997a; Kernie et al., 2000; Xu et al., 2003). Interestingly, not all areas that express TrkB are also sites of BDNF synthesis, though they may contain BDNF-positive fibers, a pattern seen in the striatum and ARC (Altar et al., 1997; Xu et al., 2003; Baydyuk et al., 2011b), indicating these areas as sites of anterograde or retrograde BDNF action, with BDNF originating from distal brain regions. In the areas that express both BDNF and TrkB, autocrine or paracrine signaling is also possible, with TrkB activation occurring from binding of BDNF released from the same or neighboring cells.

## Secretion and signaling of BDNF

BDNF is initially synthesized as a pre-pro-neurotrophin, containing a signal peptide directing sequestration of the nascent protein to the endoplasmic reticulum (ER), after which the signal peptide is cleaved, leaving proBDNF (Halban and Irminger, 1994). ProBDNF then traffics through the Golgi apparatus where it can be sorted into two different types of secretory granules, small vesicles that fuse to the plasma membrane and release their contents constitutively, or larger vesicles whose fusion to the plasma membrane is regulated and depends on Ca^2+^ levels (Lessmann et al., 2003). Intracellular cleavage of proBDNF to generate mature BDNF (mBDNF) was observed in hippocampal neurons (Mowla et al., 1999), and for a time it was believed that only secreted mBDNF was biologically active. Recent work has demonstrated that tissue plasminogen activator (tPA), a secretory protein implicated in the late phase of long-term potentiation (L-LTP), can lead to cleavage of secreted proBDNF to mBDNF by activation of plasmin, an extracellular protease (Pang et al., 2004; Yang et al., 2009). Furthermore, secretion of proBDNF is biologically relevant, as its binding to p75^NTR^ can lead to cell death and long-term depression (Teng et al., 2005; Woo et al., 2005).

BDNF functions as a homodimer that can bind to and dimerize TrkB receptors, leading to their transphosphorylation on tyrosine residues located in their intracellular domains (Patapoutian and Reichardt, 2001). This event triggers three different signaling cascades through activation of mitogen-activated protein kinase (MAPK), phosphatidylinositol 3-kinase (PI3K) and phospholipase C-gamma (PLCγ). The MAPK pathway regulates neuronal differentiation and maturation, while the PI3K pathway is essential for neuronal survival (Reichardt, 2006). In addition, signaling through the MAPK pathway leads to activation of CREB and upregulation of its target genes, as well as phosphorylation of eukaryotic initiation factor 4E (eIF4E), 4E-binding protein 1 (4E-BP1) and ribosomal protein S6, playing an important role in the regulation of protein synthesis-dependent synaptic plasticity. The PI3K pathway also is an important regulator of synaptic protein synthesis and trafficking through the activation of Akt and the mammalian target of rapamycin (mTOR), which can phosphorylate 4E-BP1 to enhance translation (Bramham and Wells, 2007). Activation of the PLCγ pathway leads to production of diacylglycerol (DAG), which can activate PKC, and inositol 1,4,5-triphosphate (IP3), which leads to the release of intracellular Ca^2+^ stores, resulting in enhancement of synaptic plasticity (Reichardt, 2006).

## Evidence for a critical role of BDNF in energy homeostasis

BDNF was first found to reduce body weight of adult rats in a study that was designed to examine the effect of chronic intracerebroventricular (icv) infusion of neurotrophins on presynaptic cholinergic function (Lapchak and Hefti, 1992). A subsequent study found that chronic injection of BDNF to the lateral ventricle of rats resulted in decreased food intake, leading to loss of body weight (Pelleymounter et al., 1995). A critical role for endogenous BDNF in the regulation of feeding behavior was demonstrated in mice with reduced BDNF levels, such as heterozygous BDNF (*Bdnf*^+/−^) mice, which had increased risk of obesity resulting from increased food intake, concomitant with elevated serum leptin and insulin levels (Lyons et al., 1999; Kernie et al., 2000). This genetic finding was confirmed and extended by the severe obesity phenotypes observed in mice expressing the TrkB receptor at ~25% of normal levels (Xu et al., 2003) and in mice where the *Bdnf* gene is deleted in neurons expressing Ca^2+^/calmodulin-dependent protein kinase II alpha (CaMKIIα) (Rios et al., 2001). Since CaMKIIα is a brain-specific protein (Lin et al., 1987), this observation demonstrates that BDNF acts on neurons of the central nervous system to affect energy balance.

Deficiencies in BDNF signaling are also associated with obesity in humans. A *de novo* missense mutation in a key tyrosine residue of the TrkB kinase domain or loss of a functional *Bdnf* allele has been found to be associated with hyperphagia, severe obesity, and impaired cognitive function in children (Yeo et al., 2004; Gray et al., 2006). A subgroup of patients with the Wilms' tumor, aniridia, genitourinary anomalies, and mental retardation (WAGR) syndrome display hyperphagia and obesity (Fischbach et al., 2005). The WAGR syndrome results from heterozygous, variably sized, contiguous deletions on chromosome 11, which extend into the *Bdnf* gene in some cases. Careful mapping of the deletion regions in 33 patients with the WAGR syndrome has identified haploinsufficiency of BDNF as the cause of the accompanied obesity disorder (Han et al., 2008). Furthermore, large-scale genome-wide association studies link a common single nucleotide polymorphism in the *Bdnf* gene, rs6265, to human obesity (Hotta et al., 2009; Thorleifsson et al., 2009), and rs6265 has been shown to be associated with human eating disorders (Ribases et al., 2004; Gratacos et al., 2007). This polymorphism changes the Val residue at position 66 of pre-pro-BDNF to a Met residue and impairs activity-dependent BDNF secretion through the regulated secretory pathway (Egan et al., 2003). Additional evidence of a role for BDNF in obesity comes from a knock-in mouse strain, which contains the Val66Met mutation and exhibits increased body weight (Chen et al., 2006).

## BDNF as an anorexigenic factor

BDNF plays crucial roles in the development and maintenance of neural circuits (Xu et al., 2000b; Huang and Reichardt, 2001; Baydyuk et al., 2011a). Thus, obesity syndrome induced by BDNF insufficiency could result from either diminished anorexigenic activity of BDNF or structural defects in neural circuits that are important for the control of energy homeostasis. The severe obesity phenotype in a mouse mutant where the *Bdnf* gene is deleted in many brain regions using a *Cre* transgene under the control of the CaMKIIα promoter (Rios et al., 2001) has been widely cited as evidence for BDNF as an anorexigenic factor because the transgene starts to express Cre recombinase in the hippocampus during the third postnatal week, after many developmental events in the brain have already been completed (Tsien et al., 1996; Xu et al., 2000a). However, CaMKIIα is expressed in the hypothalamus as early as the first postnatal week (An and Xu, unpublished data), when many hypothalamic neurons are still sending their axons to targets (Bouret et al., 2004), suggesting that the development of hypothalamic neural circuits could be altered in this conditional *Bdnf* knockout. Indeed, one study showed that the projection of POMC-expressing neurons in the ARC to the DMH was impaired in one *Bdnf* mutant strain (Liao et al., 2012). More supportive evidence for BDNF as an anorexigenic factor comes from the observation that selective deletion of the *Bdnf* gene in the VMH and DMH of adult mice using Cre-expressing virus still induced hyperphagia and increased weight gain (Unger et al., 2007), although there is still the possibility that the *Bdnf* deletion leads to degeneration of an anorexigenic neural circuit.

If BDNF is acting as a mediator of signals regulating energy balance, its expression levels should change to reflect nutritional status. Two-day food deprivation was found to drastically reduce levels of *Bdnf* mRNA in the mouse VMH, without affecting *Bdnf* gene expression in the cerebral cortex (Xu et al., 2003). This observation not only demonstrates that BDNF is a bona fide anorexigenic factor, but also indicates the importance of VMH BDNF in the control of energy homeostasis. Furthermore, food deprivation was also found to reduce BDNF protein levels in the dorsal vagal complex (DVC) (Bariohay et al., 2005). Whether food deprivation alters *Bdnf* gene expression in the DMH, LH, and PVH remains to be determined. Melanocortin and glucose are likely key mediators linking energy status to *Bdnf* gene expression in the VMH, as administration of either a melanocortin analog or glucose into fasted mice increased levels of *Bdnf* mRNA in the VMH (Xu et al., 2003; Unger et al., 2007). The *Bdnf* gene is expressed from multiple promoters in the VMH (Tran et al., 2006; Unger et al., 2007), and glucose regulates *Bdnf* gene expression in the VMH through the exon I promoter (Unger et al., 2007). One study reported that systemic leptin administration increased levels of *Bdnf* mRNA and BDNF protein in the VMH (Komori et al., 2006). Since BDNF neurons in the VMH do not express the leptin receptor (Liao et al., 2012), leptin may indirectly regulate *Bdnf* gene expression in the VMH.

Collectively, these studies show that BDNF levels in some brain regions change in response to nutritional status and that postnatal ablation of BDNF expression leads to hyperphagia and obesity. These biochemical and genetic findings, along with the pharmacological observation that icv infusion of BDNF reduces food intake and body weight (Lapchak and Hefti, 1992; Pelleymounter et al., 1995), strongly indicate that BDNF acutely and actively regulates energy homeostasis. Given the crucial role of BDNF in neuronal development, it is conceivable that developmental defects in neural circuits also contribute to the massive obesity observed in mice with impaired BDNF activity. Interestingly, central infusion of BDNF was found to reverse hyperphagia and excessive weight gain in *Bdnf*^+/−^ mice (Kernie et al., 2000). This result suggests that developmental defects in neural circuits relevant to feeding are not permanent in *Bdnf* or *TrkB* mutants and could be overcome in later life by increasing BDNF activity.

## Roles of BDNF in the regulation of energy expenditure

A decrease in energy expenditure can lead to obesity, and one of key forms of energy expenditure is thermogenesis. In mouse and rat, the interscapular brown adipose tissue (IBAT) is the major site of thermogenesis. Sympathetic inputs from the stellate ganglion increase the expression of uncoupling protein 1 (UCP1) in IBAT, which allows the energy generated from β oxidation of fatty acids to dissipate as heat in response to physiological stimuli such as cold exposure and overeating (Cannon and Nedergaard, 2004; Clapham, 2012). Some neurons in several hypothalamic areas, including DMH, LH, PVH, and retrochiasmatic area, are poly-synaptically connected to IBAT, as revealed by pseudorabies virus retrograde tracing (Oldfield et al., 2002; Cano et al., 2003).

Both peripheral and icv administration of BDNF were found to increase turnover of norepinephrine and *UCP1* gene expression in IBAT and to restore thermogenesis in food-deprived *db*/*db* mice (Nonomura et al., 2001; Tsuchida et al., 2001). Direct injection of BDNF to the PVH and VMH in rats resulted in an increase in energy expenditure and a decrease in weight gain (Wang et al., 2007b, 2010). However, BDNF action in these two sites is not the same, as BDNF injection to the PVH was found to increase the expression of UCP1 in IBAT without affecting spontaneous physical activity (Wang et al., 2007b), whereas BDNF injection to the VMH increased spontaneous physical activity without altering UCP1 expression in IBAT (Wang et al., 2010). These pharmacological studies suggest that enhancing TrkB signaling in the PVH and VMH increases energy expenditure by either directly or indirectly modulating hypothalamic neurons that are connected to IBAT and the motor system, respectively. Interestingly, it was found that environmental enrichment increased thermogenesis by inducing the appearance of cells with prototypical brown fat morphology and high UCP1 levels in white fat tissue, and that BDNF mediated this effect of environmental enrichment (Cao et al., 2011). This observation suggests that endogenous BDNF also regulates energy expenditure, although it remains unknown if environmental enrichment affects thermogenesis in IBAT as well.

Genetic data collected from *Bdnf* mouse mutants has not painted a clear picture regarding the role of BDNF in energy expenditure. Pair feeding was found to correct excessive weight gain in *Bdnf* heterozygous mice (Coppola and Tessarollo, 2004), mice where the *Bdnf* gene was selectively deleted in the DMH and VMH (Unger et al., 2007), and mice lacking the long isoform of *Bdnf* mRNA (Liao et al., 2012). These studies indicate that the total energy expenditure in these mutants remains unchanged. This evidence shows a dominant role of hyperphagia in the development of obesity when BDNF signaling is impaired; however, it is not sufficient to rule out a role for BDNF in the regulation of energy expenditure. *Bdnf* heterozygous mice showed elevated locomotor activity (Kernie et al., 2000), which may compensate for reduced thermogenesis and/or reduced resting metabolic rate. It is possible that BDNF derived from the short isoform of *Bdnf* mRNA in a brain region other than the DMH and VMH may regulate thermogenesis and resting metabolism.

## Production sites of the BDNF protein crucial for energy homeostasis

The VMH is likely a site for the synthesis of BDNF protein that is critical for energy homeostasis. The *Bdnf* gene is expressed in many neurons in the ventrolateral and dorsomedial parts of the VMH, and its mRNA levels in this area are among the highest in the brain (Xu et al., 2003). Food deprivation was found to selectively and drastically reduce levels of *Bdnf* mRNA in the VMH (Xu et al., 2003), and administration of melanocortin agonists or glucose was able to partially reverse this reduction in *Bdnf* gene expression (Xu et al., 2003; Unger et al., 2007). This observation suggests that hunger suppresses *Bdnf* gene expression in the VMH, whereas feeding, which is accompanied by an increase in melanocortin signaling and glucose levels in the brain, does the opposite. This inference would predict that deletion of the *Bdnf* gene in the VMH should lead to hyperphagia and obesity. Deletion of the *Bdnf* gene in the VMH and DMH of adult mice with adeno-associated virus (AAV) expressing Cre recombinase indeed caused modest hyperphagia and obesity (Unger et al., 2007). However, this result does not completely address the role of VMH BDNF in the control of energy homeostasis, because the obesity phenotype in this mutant was very subtle in comparison to mutant mice where the *Bdnf* gene was deleted in many brain regions or where TrkB was systemically down-regulated (Rios et al., 2001; Xu et al., 2003). The subtlety of the obesity phenotype is likely due to incomplete AAV-mediated *Bdnf* deletion in the VMH. It may also result from the late onset of the *Bdnf* deletion, which would remove the impact of BDNF deficiency on neuronal development. When a *Cre* transgene driven by the promoter for steroidogenic factor 1 (*SF1-Cre*) was used to delete the *Bdnf* gene in SF1-expressing VMH neurons, the resulting mutant mice did not display any body weight phenotype (Dhillon et al., 2006). Given that SF1 and BDNF are only partially co-expressed in the VMH (Tran et al., 2003), this is not a surprising result. Development of additional VMH-specific *Cre* transgenic mice will greatly facilitate elucidation of the role of VMH BDNF in the control of feeding behavior and body weight.

Food deprivation also decreased levels of BDNF protein in the rat DVC, whereas refeeding or peripheral injection of the anorexigenic hormones leptin or CCK increased BDNF protein levels in this area (Bariohay et al., 2005). This observation suggests that BDNF in the DVC may regulate food intake. However, it remains to be determined whether BDNF in the DVC is synthesized locally or comes from other brain regions through retrograde or anterograde transport. If the BDNF protein is locally generated, it would be important to investigate in which neuronal populations refeeding and anorexigenic factors act to regulate *Bdnf* gene expression.

Mice in which *TrkB* gene expression was reduced to a quarter of the normal amount, or the *Bdnf* gene was deleted in CaMKIIα-expressing neurons, displayed markedly increased food intake on high fat diet compared to low fat diet (Xu et al., 2003; Cordeira et al., 2010). This observation indicates that BDNF is involved in hedonic feeding in response to palatable diet, in addition to its role in the homeostatic regulation of energy balance in response to fat stores and peripheral signals of nutrient status. This hedonic phenotype is dependent on the action of VTA-derived BDNF in the NAc, since consumption of high fat diet decreased *Bdnf* mRNAs in the VTA and deletion of the *Bdnf* gene in the VTA induced hyperphagia on high fat diet but not low fat diet (Cordeira et al., 2010).

Neurons produce two forms of *Bdnf* mRNA encoding the same protein due to the presence of two alternative polyadenylation sites: short 3′ UTR *Bdnf* mRNA and long 3′ UTR *Bdnf* mRNA. One study indicates that BDNF protein regulating food intake is translated from long 3′ UTR *Bdnf* mRNA, likely in dendrites (Liao et al., 2012). This study used a mouse mutant with multiple SV40 polyadenylation signal sequences inserted shortly downstream of the first *Bdnf* polyadenylation site, such that the long *Bdnf* 3′ UTR is truncated (Gorski et al., 2003; An et al., 2008). The mutant mice were found to lack dendritic *Bdnf* mRNA (An et al., 2008) and developed severe hyperphagic obesity (Liao et al., 2012). Importantly, early viral expression of long, but not short, 3′ UTR *Bdnf* mRNA in the VMH and nearby regions completely prevented these animals from developing hyperphagia and obesity (Liao et al., 2012). It remains unknown, however, in which specific neuronal populations BDNF is translated from long 3′ UTR *Bdnf* mRNA to regulate appetite.

## Action sites of BDNF to control energy homeostasis

Not much is known about the action sites of BDNF with regard to its role in the control of energy homeostasis. BDNF exerts its biological effects through two receptors, TrkB and p75^NTR^ (Reichardt, 2006). Since mutations in the gene for TrkB, but not p75^NTR^, lead to obesity in humans and mice (Lee et al., 1992; Xu et al., 2003; Yeo et al., 2004), BDNF should act on the TrkB receptor to mediate its effects on appetite and energy expenditure. BDNF produced in a neuron can reach TrkB-expressing neurons through four modes (DiStefano et al., 1992; von Bartheld et al., 1996; Altar et al., 1997): being released and binding to TrkB on the same neuron (autocrine) or neighboring neurons (paracrine); being anterogradely transported to the axonal terminals of the neuron and released to bind TrkB on targeted neurons (anterograde); and being retrogradely transported to cell bodies of innervating neurons following release and binding to TrkB on innervating axonal terminals (retrograde). Because of the complexity of BDNF action and the widespread expression of BDNF and TrkB in the brain, it is a challenge to identify TrkB-expressing neuron populations that mediate the effects of BDNF on energy homeostasis.

Pharmacological studies have suggested several putative sites where BDNF acts to control energy homeostasis. Direct injection of BDNF into either the PVH or the VMH of adult rats was found to decrease food intake and increase energy expenditure (Wang et al., 2007a,b,c, 2010). Delivery of BDNF into the medial NTS of the brainstem also reduced food intake and increased thermogenesis, and these effects could be blocked with a TrkB antagonist (Spaeth et al., 2012). It remains to be determined whether endogenous BDNF at these anatomical sites also has similar effects on energy homeostasis. The PVH, VMH, and NTS are complex structures and contain many distinct neuronal populations. It is important to determine on which populations of TrkB-expressing neurons BDNF acts to regulate energy homeostasis. These questions can be addressed using conditional *TrkB* mouse knockouts. Once the neuronal populations are identified, researchers can focus on the mechanisms by which BDNF modulates the function of these neurons.

In addition to the hypothalamus and the brainstem, the mesolimbic dopamine system may be another target for BDNF to affect food intake, especially hedonic eating. Consumption of high fat diet was found to increase *TrkB* mRNA in the VTA (Cordeira et al., 2010). Mice with impaired BDNF signaling exhibited much more severe hyperphagia on fat-rich diet than on low-fat diet (Xu et al., 2003; Cordeira et al., 2010). Mice lacking BDNF synthesis in CaMKIIα-expressing neurons displayed hypersensitivity of the dopamine receptor D_1_, such that peripheral injection of a D_1_ receptor agonist normalized the hyperphagia on high-fat diet (Cordeira et al., 2010). The effect of BDNF deficiency on the dopamine receptor D_1_ is likely indirect, as the vast majority of TrkB-expressing neurons are striatal medium-sized spiny neurons that express the dopamine receptor D_2_ (Baydyuk et al., 2011b). This indirect effect may be a compensatory response to decreased evoked release of dopamine in the NAc and the dorsal striatum. As the VTA has been identified as an important source of BDNF contributing to regulation of hedonic eating (Cordeira et al., 2010), it would be interesting to determine whether BDNF acts on TrkB-expressing neurons in the VTA or the nucleus accumbens to regulate consumption of palatable food. It is critical to understand the changes in the structure and function of the neural circuitry for reward when BDNF signaling is impaired, which leads to hyperphagia on high-fat diet, since excessive intake of high fat diet is likely an important cause of the obesity pandemic in humans.

It is worth noting that one study reported that while central administration of TrkB agonists was anorexigenic in non-human primates, similar to what has been observed for rodents, peripheral administration was orexigenic, opposite to what is observed for rodents (Lin et al., 2008). Although the observation has to be reproduced, this brings into question the assumption that BDNF regulates energy balance solely through activation of central pathways. One possible peripheral BDNF target might be the vagal afferents (Fox et al., 2012).

## Direct regulation of glucose homeostasis by BDNF

Obesity in mice with BDNF deficiency is associated with hyperglycemia and impaired glucose tolerance (Kernie et al., 2000; Rios et al., 2001; Liao et al., 2012), and this glucose phenotype is at least in part attributable to the direct action of BDNF on glucose homeostasis. In several animal models of obesity and diabetes, including *db*/*db* mice, diet-induced obesity mice and lethal yellow agouti mice, peripheral administration of BDNF has been shown to prevent or ameliorate the diabetic and obese phenotypes (Nakagawa et al., 2003; Yamanaka et al., 2008). In *db*/*db* mice, BDNF could potentiate the action of insulin in peripheral tissues, reducing serum glucose in obese, diabetic *db*/*db* mice (Ono et al., 1997). The requirement for insulin to mediate the hypoglycemic effect of peripheral BDNF was demonstrated in streptozotocin-induced type 1 diabetic mice, which had no reduction in blood glucose levels after BDNF treatment alone, but showed an increased hypoglycemic action of insulin when co-administered with BDNF (Nakagawa et al., 2000). Peripheral BDNF administration lead to decreased fed blood glucose levels in obese diabetic *db*/*db* mice by affecting blood glucose control, rather than food intake. BDNF administration also normalized fasted blood glucose levels, possibly through reduced hepatomegaly (Tonra et al., 1999). Even intermittent peripheral administration of BDNF was effective at reducing blood glucose levels in obese diabetic *db*/*db* mice, suggesting a long-lasting effect of BDNF on the control of glucose metabolism (Ono et al., 2000). The lower levels of pancreatic insulin and elevated levels of glucagon in hyperglycemic *db*/*db* mice were restored to normal levels by BDNF treatment, with increased islet beta-cell area in BDNF-treated mice and a reversal of the decrease in pancreatic secretory granules, indicating a role for peripheral BDNF in restoring impaired pancreatic insulin production and secretion in *db*/*db* mice (Nakagawa et al., 2000; Yamanaka et al., 2006).

CNS neurons may mediate some effects of peripheral BDNF administration on glucose homeostasis. Chronic subcutaneous BDNF administration in *db*/*db* mice led to increased insulin receptor activation in the liver and insulin-stimulated PI3K activity in the liver, skeletal muscle and IBAT. However, no direct effect of BDNF was found on cultured hepatocytes, L6 muscle cells or 3T3-L1 adipocytes, suggesting an indirect route of action. This may possibly be through regulation of central mechanisms leading to peripheral signaling, as suggested by the observation that centrally administered BDNF also had hypoglycemic effects, and led to similar increases in liver insulin receptor activation and insulin-stimulated PI3K activity (Nakagawa et al., 2000; Tsuchida et al., 2001). Peripheral administration of BDNF increased glucose uptake and norepinephrine content in muscle and IBAT of *db*/*db* mice, indicating the activation of sympathetic pathways to regulate glucose metabolism (Yamanaka et al., 2007). It remains unclear how peripherally administered BDNF gets into the CNS and which CNS neurons BDNF acts on to regulate glucose uptake through the sympathetic nervous system.

## Interaction of BDNF-TrkB signaling with other anorexigenic factors

Several studies indicate that BDNF acts downstream of anorexigenic factors such as leptin and CCK to regulate food intake and body weight. Peripheral leptin administration was found to increase *Bdnf* mRNA levels in the VMH (Komori et al., 2006) and BDNF protein levels in the DVC (Bariohay et al., 2005). Peripheral CCK injection also increased BDNF protein levels in the DVC and hypothalamus (Bariohay et al., 2005). These effects on BDNF protein levels likely result from increased translation of *Bdnf* mRNA in dendrites, as leptin increased dendritic *Bdnf* mRNA translation in cultured hypothalamic neurons (Liao et al., 2012). In support of this argument, CCK increased BDNF protein levels in the DVC within 30 min (Bariohay et al., 2005), a duration that may be too short for regulation at the transcriptional level. Leptin exerts its physiological action through the long-form leptin receptor LepRb (Chua et al., 1996; Lee et al., 1996). Interestingly, BDNF-expressing neurons in the DMH and VMH do not express LepRb, but leptin administration was found to induce expression of c-Fos in these neurons (Liao et al., 2012). This observation indicates that leptin indirectly stimulates synthesis of *Bdnf* mRNA and BDNF protein, likely by activating BDNF-expressing neurons through a polysynaptic neural circuit.

BDNF signaling through the TrkB receptor is necessary for the anorexigenic action of leptin. Peripheral leptin injections reduced food intake in wild-type mice, but failed to do so in mice lacking long 3′ UTR *Bdnf* mRNA (Liao et al., 2012). Leptin administration into the mNTS also reduced food intake, and co-administration of a TrkB antagonist attenuated the intake-suppressive effect of leptin (Spaeth et al., 2012). Activation of LepRb by leptin induces phosphorylation of signal transducer and activator of transcription protein 3 (STAT3) (Munzberg et al., 2003). In the DMH and VMH of mice and rats, leptin activates STAT3 and induces c-Fos expression in distinct neuronal populations (Hubschle et al., 2001; Liao et al., 2012), indicating that LepRb-expressing neurons send inputs to non-LepRb-expressing neurons and subsequently induce c-Fos expression in these cells. In mice lacking long 3′ UTR *Bdnf* mRNA, leptin injection activated LepRb normally in the ARC, DMH, and VMH, as indicated by phosphorylation of STAT3; however, leptin-induced c-Fos expression was impaired in the ARC and VMH and abolished in the DMH (Liao et al., 2012). These observations indicate that one way by which BDNF controls energy homeostasis is to regulate the formation, maintenance, and/or function of neuronal connections. When BDNF signaling is compromised, neural circuits in these hypothalamic areas are dysfunctional and fail to transmit the anorexigenic signal of leptin, leading to leptin resistance and obesity.

BDNF also interacts with the melanocortin system to regulate energy homeostasis. The initial consideration of this interaction arose from the appreciation of similar obesity phenotypes between *TrkB* mutant mice and *Mc4r*^−/−^ knockout mice (Xu et al., 2003). Both mutants exhibit increased linear growth, more severe obesity in females than in males, and extremely increased appetite on fat-rich diet (Huszar et al., 1997; Butler et al., 2001; Xu et al., 2003). Furthermore, BDNF infusion into the lateral ventricle suppressed the hyperphagia and excessive weight gain observed on fat-rich diet in agouti lethal yellow mice that overexpress the agouti protein, an antagonist of melanocortin receptors (Xu et al., 2003). Additional evidence for BDNF acting downstream of the MC4R was obtained from *TrkB*^*F*616*A*^ mice in which the phenylalanine residue at position 616 of the TrkB receptor is changed to an alanine residue, which makes the TrkB kinase sensitive to inhibition by the small molecule 1NaPP1 (Chen et al., 2005). Systemic administration of 1NaPP1 in these mice abolished the anorexigenic effect of a selective MC4R agonist (Bariohay et al., 2009). Melanocortin agonists were found to increase *Bdnf* mRNA levels in the VMH (Xu et al., 2003), to increase BDNF protein levels in the DVC (Bariohay et al., 2009), and to stimulate BDNF release from hypothalamic slices (Nicholson et al., 2007). Given that neuronal activity stimulates gene expression and release of BDNF (Waterhouse and Xu, 2009), MC4R signaling may enhance TrkB activity by either directly or indirectly stimulating BDNF-expressing neurons. α-MSH acts on the MC4R mainly in the PVH and the intermediolateral cell column of the thoracic spinal cord to regulate food intake and energy expenditure (Balthasar et al., 2005; Rossi et al., 2011), and thus further studies are needed to investigate the interaction between the MC4R and the BDNF-TrkB pathway in these two nuclei.

## Summary and perspective

Strong evidence of a crucial role for BDNF in the control of energy homeostasis has been accumulated from genetic and pharmacological studies during the last decade or so. The Val66Met polymorphism in the *Bdnf* gene is a common allele (Shimizu et al., 2004) and has been linked to human obesity (Hotta et al., 2009; Thorleifsson et al., 2009). It is likely that BDNF acts downstream of many anorexigenic factors to control body weight. Thus, elucidation of the mechanisms by which BDNF controls appetite and energy expenditure should have important implications for human health. Recent studies indicate that synaptic plasticity in the hypothalamus plays a crucial role in its control of energy homeostasis (Pinto et al., 2004; Yang et al., 2011; Liu et al., 2012). Given its important role in synaptic plasticity, BDNF may regulate energy homeostasis by modulating synaptic plasticity in relevant areas. With the availability of more and more *Cre* transgenic mice and circuitry-mapping tools, it is becoming feasible to identify the neural circuits mediating the effects of BDNF on energy homeostasis. Identification of these circuits and elucidation of the mechanisms by which BDNF acts on them will greatly enhance our understanding of human obesity and hopefully bring us closer to a strategy for designing effective drug treatments for this disease.

### Conflict of interest statement

The authors declare that the research was conducted in the absence of any commercial or financial relationships that could be construed as a potential conflict of interest.
